# Study on Physio-chemical Properties of plasma polymerization in C_2_H_2_/N_2_ plasma and Their Impact on COL X

**DOI:** 10.1038/s41598-017-09747-4

**Published:** 2017-08-22

**Authors:** Samaneh Ghafouri, Sara Abdijahed, Shirin Farivar, Seyed Iman Hosseini, Fatemeh Rezaei, Abdolreza Ardeshirylajimi, Babak Shokri

**Affiliations:** 1 Laser-Plasma Research Institute, University of Shahid Beheshti,G.C, Tehran, Iran; 2 Cellular and Molecular Biology, Shahid Beheshti University, G.C, Tehran, Iran; 3 Faculty of Biological Sciences, Shahid Beheshti University, G.C, Tehran, Iran; 4 Department of Physics, Shahrood University of technology, Shahrood, Iran; 5 Research Unit Plasma Technology (RUPT), Department of Applied Physics, Faculty of Engineering & Architecture, Ghent University, St-Pietersnieuwstraat 41 B4, 9000 Ghent, Belgium; 6 Department of Tissue Engineering and Regenerative Medicine, School of Advanced Technologies in Medicine, Shahid Beheshti University of Medical Sciences, Tehran, Iran; 7 Department of Physics, University of Shahid Beheshti, G.C., Tehran, Iran

## Abstract

Nitrogen-containing plasma polymerization is of considerable interest for tissue engineering due to their properties on cell adhesion and mesenchymal stem cells (MSCs) response. In this study, low-pressure RF plasma of acetylene and nitrogen was used to deposit nitrogen-containing plasma polymerized coatings on several substrates. Deposition kinetics and surface characteristics of coatings were investigated in terms of RF power and gas flow ratio. OES was used to monitor the plasma process and investigate the relation between the film structure and plasma species. Presence of several bonds and low concentration of amine functional groups were determined using FTIR and Colorimetric methods. Contact angle goniometry results indicated about 30% increase in surface hydrophilicity. Stability of coatings in air and two different liquid environments was examined by repeating surface free energy measurements. Deposited films exhibited acceptable stability during the storage duration. Surface roughness measured by AFM was found to decrease with growing concentration of nitrogen. The deposition rate increased with increasing RF power and decreased with growing concentration of nitrogen. Zeta potential measurements of coatings revealed the negative potential on the surface of the thin films. Temporary suppression of collagen X in the presence of plasma coatings was confirmed by RT-PCR results.

## Introduction

The use of plasma technology in the context of plasma medicine for wound healing, sterilization of various surfaces, inactivation of pathogens in contact with living tissues, selective treatment of cancer cells, and also for dentistry and dermatological applications is a growing important research area^1–5^. One of plasma deposition process, known as plasma polymerization is a process for the formation of polymeric materials under plasma conditions and capable to produce films with thickness ranging from 50 A to 2 µm. In this process the radicals are building blocks for polymeric layers^6^. Plasma polymers different characteristics in comparison to the conventional polymers. However, in both cases the characteristics of the polymers are dependent on the chemical properties of the monomers. Compared to the common polymerization techniques, which specific monomers are required, the plasma polymerization process can be performed using all types of organic compounds^7^. Thin plasma polymerized (PP) films have considerable biomedical applications.

**Figure 1:**
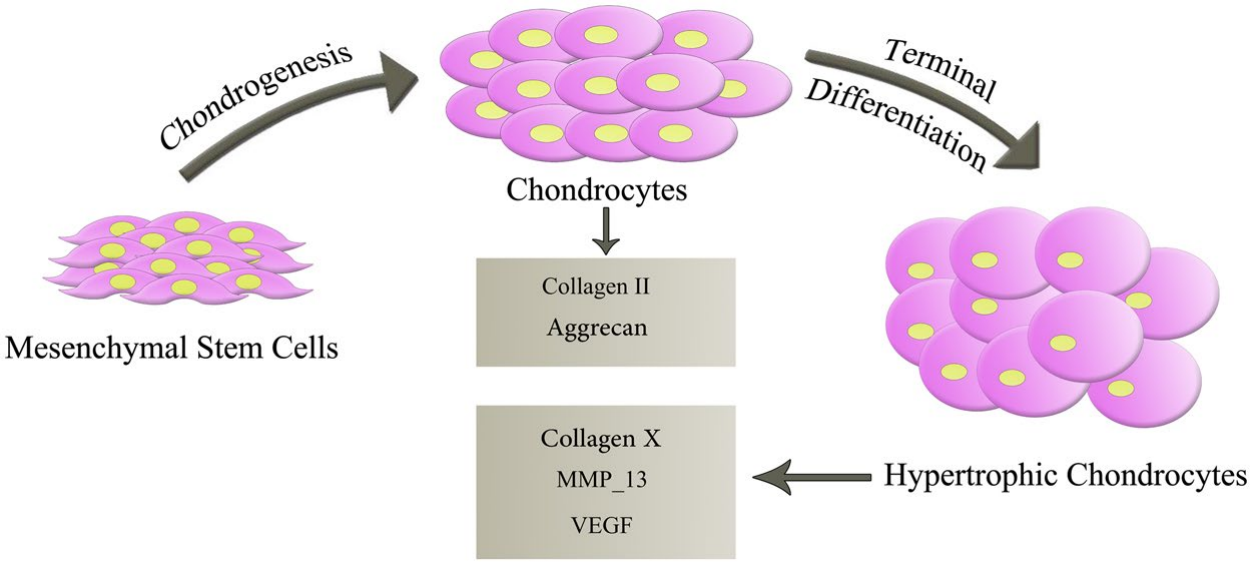
Schematic presentation of cartilage life cycle.

Articular cartilage damage, especially in older people, is a common problem which many patients suffer from^8, 9^. In fact, traumatic injuries and age-related diseases associated with cartilage, are a major health problem in the world^10, 11^. Most probably, the lack of blood supplies and subsequently presence of undifferentiated cells at the site of injury play the main role in restriction of repair response of articular cartilage^12–14^. This is invoked according to the biological characteristics of cartilage tissue that limits its capacity for self-renewal^15^. During the recent years, the use of mesenchymal stem cells (MSCs) has been gained much interest because of their differentiation capabilities to some new tissues and its immunomodulatory properties^16–18^. MSCs are multifunctional cells that have potential of changing to some kind of body tissues such as, cartilage, bone, muscle, tendon, ligament, fat and so on. One of distinct problems with current cartilage repair techniques is that MSCs from osteoarthritis (OA) patients rapidly express type X collagen (COLX), a marker of late stage chondrocyte hypertrophy associated with endochondral ossification; consequently they will not be able to be used effectively in treatments as a source of autologous stem cells. Recently, it has been reported that nitrogen-containing plasma-polymer films would affect the expression of COL X. Three functionally and structurally distinct stages are considered for cartilage life cycle, namely the resting zone, proliferative zone, and hypertrophic cartilage zone^19^ (Fig. 1). The chondrocytes in all three zones are embedded in a cartilaginous extracellular matrix (ECM).

In the resting zone, chondrocytes act as proliferating cells and lie as single cells or in small clusters in a dense ECM, which express type II collagen and proteoglycans. The proliferating chondrocytes display regular columns and they divide rapidly. They synthesize matrix proteins including, collagens II, IX, and XI, cartilage specific proteoglycans, etc. After a finite number of cell divisions, chondrocytes start to enlarge by partly resorb their ECM, as they mature to the hypertrophic phenotype and express COL X. The transition between the proliferative and hypertrophic zones occurs when proliferative chondrocytes start to round up and begin to enlarge. In the hypertrophic zone, the ECM is rich in COL X and the matrix starts to mineralize in the zone of provisional calcification. This coordinated proliferation and differentiation of growth plate chondrocytes is required for normal growth and development of the skeleton^20^.

In recent years, there has been an increasing attentions to study on the differentiation of MSCs from patients with osteoarthritic (OA). It is due to autologous stem cells requirement of these patients if biological repair of cartilage is to be a therapeutic option^21, 22^. However, the main downside of current cartilage and disc tissue engineering is rapid expression of type X collagen in human MSCs derived from OA patients^23^. Some studies have been conducted to suppress COL X using growth factors, however, until now no reliable report on the possible effect of the substratum on chondrocyte hypertrophy is available. Immobilization of bio-molecules and also colonization of surfaces of living cells are only a few examples of the vast applications of biotechnology for thin PP films.

In 2005, Nelea *et al*.^24^ investigated the effect of thin plasma-polymer films, known as nitrogen rich plasma-polymerized ethylene (PPE:N), on the behavior of COL X In a period of 14 days. They used ethylene and ammonia gases to deposit the films by an RF-PECVD coating process. They concluded that these films largely inhibited collagen X expression, especially in the seventh day. Another work was done by Petit *et al*. in 2010^20^, in which they reported the same result as Nelea *et al*. However, Petit *et al*. used ethylene and nitrogen gases in an atmospheric pressure dielectric barrier discharge (DBD) plasma. In 2011, Rampersad *et al*.^25^ conducted a study to determine if the suppression of COL X by PPE:N is maintained when MSCs are transferred to pellet cultures in serum-free media. They used both low- and high-pressure plasma systems for synthesis of PPE:N films. Their results confirmed the potential of both low- and high- pressure-PPE:Ns (L- and H- PPE:N) in suppressing COL X expression. Also, they showed that when MSCs were transferred to pellet cultures, the expression level of COL X was further decreased by pre-incubation on H-PPE:N. They concluded that these plasma coatings are promising for tissue engineering of cartilage and disc tissues. To deposit L-PPE:N and H-PPE:N films, Rampersad *et al*. also used ethylene/ammonia and ethylene/nitrogen mixtures, respectively.

According to the importance of this new research field, further investigations are required to assess the effects of surface chemistry versus surface morphology in the mechanism of COL X suppression using PPE:N films. Physio-chemical properties of the thin films including roughness, hydrophobicity, zeta potential, surface charge, and active functional groups govern factors in the biological environments^26^. However, it was not well understood which mechanisms control MSCs differentiation^23^. Several papers reported that nitrogen functional groups (especially amine groups) influence the MSCs response and collagen-10 suppression^24, 27–29^ as an important hypertrophy and osteogenesis marker via the inhibition of the specific cyclooxygenase-1 (COX-1: SC-560). It is speculated that nitrogen functional groups work by deactivating the “hedgehog signaling” pathway involved in the development of bone cells. In the other hands, protonated amine (NH_2_) groups among the nitrogen functional groups have a localized positive charge that can absorb biomolecules with negatively charged in the aqueous solution and are proffered at physiological PH^26^.

According to the importance of the cartilage damages, the present paper provides comprehensive analysis on the application of PP films in tissue engineering. In addition, as there are no sufficient studies on the deposition of acetylene as hydrocarbon source, we have used this type of hydrocarbon in this paper.

In this study, we used (acetylene/nitrogen gas mixture) for synthesis of L-PPA:N films by RF-PECVD technique. Firstly, the plasma characteristics and reactive plasma species were investigated. The surface physical and chemical properties were also studied due to their importance in determining the response of MSCs in contact with the surfaces. We studied surface potential, wettability, surface free energy, morphology, and also available chemical bonds on the surface. Additionally, the stability of the surface properties after deposition was determined by aging study in two different liquid media (deionized water and culture medium) and also in air. Finally, the response of MSCs and expression of COL X, under the influence of plasma coatings, were studied.

## Results and Discussion

### Plasma Diagnostics

The active species generated during the discharge were detected with OES. Due to atomic excitations in the plasma environment and optical emissions in various frequencies, the emission spectra with different intensities were observed in the conducted experiments. Increasing intensity of spectra is related to more excitations which occur during the process.

**Figure 2:**
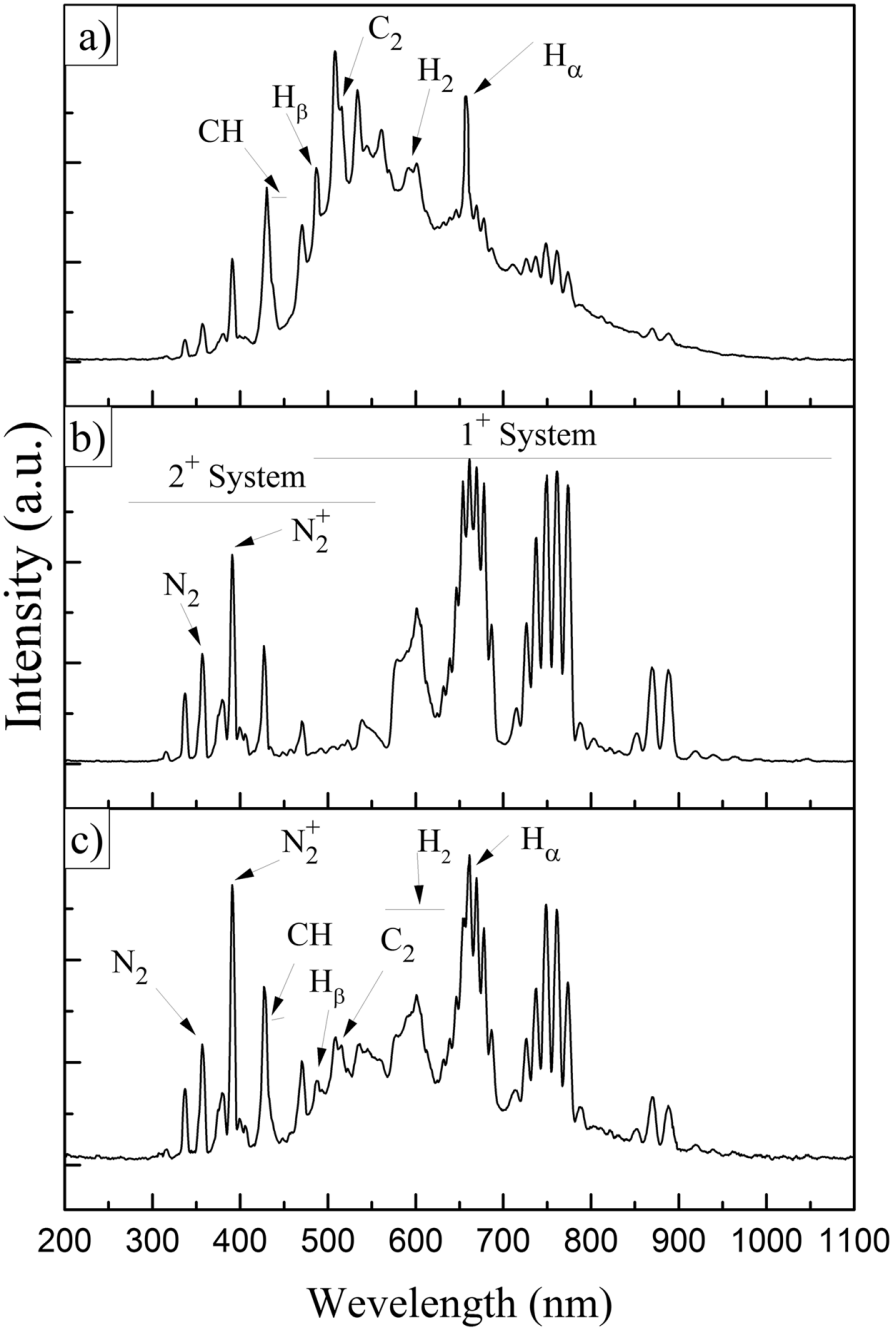
Optical emission spectra of the discharges in (**a**) pure acetylene (P = 20 w, 20 sccm flow rate), (**b**) pure nitrogen (P = 20 w, 40 sccm flow rate), and (**c**) gas mixture (P = 20 w, R = 2).

As a consequence, more intensified emission and more active plasma environment we will have. Typical representative OES spectra for discharges in pure acetylene, pure nitrogen and gas mixture of these two gases are displayed in Fig. 2 and Table 1 lists the main active contributions and their electronic transitions.

**Table 1:** Summary of main species detected by OES^51, 63–65^.

Species	Wavelength (nm)	Electronic transition
${H}_{\alpha }$	656.5	$3{d}_{2}{D}_{3/2}-2{p}_{2}{P}_{3/2}^{0}$
${H}_{\beta }$	486.8	$4{d}^{2}{D}_{3/2}^{0}-2{p}^{2}{P}_{1/2}^{0}$
$CH$	430.3	${A}^{2}\Delta -\,{X}^{2}\Pi \,(0,0)$
${N}_{2}$	356.8	${C}^{3}{\Pi }_{u}-\,{B}^{3}{\Pi }_{g}\,(0,1)$
${N}_{2}^{+}$	390.8	${B}^{2}{\Sigma }_{u}^{+}-\,{X}^{2}{\Sigma }_{g}^{+}\,(0,0)$
${H}_{2}$	580–650	$(Fulcher\,\alpha ){A}^{2}\Pi -\,{X}^{2}\Sigma$
${C}_{2}$	515.9	${A}^{3}{\Pi }_{R}-\,{X}^{\text{'}3}{\Pi }_{u}\,(0,0)$

There are only some specific species which are observed in the visible spectra of hydrocarbon plasma. Acetylene is commonly used for deposition of diamond like films^30^. The most intense transition lines in the pure acetylene spectrum are related to *CH*, ${H}_{\alpha }$, ${H}_{\beta }$, ${H}_{2}$ and $C2$ (Fig. 2a). It seems that *C*_2_ and *CH* radicals play important role in growth mechanism of the films^31, 32^. Among different reactions which lead to production of *CH* radical in the acetylene plasma, electron impact induced dissociation of acetylene is the most notable reaction:

1 $${C}_{2}{H}_{2}+e\,\to 2CH+e$$

The first and second positive systems of pure nitrogen plasma can be observed in Fig. 2b. Although the first negative, fourth positive and fifth positive systems, as well as Vegard-Kaplan bands observed in spectra^33, 34^, we have not reported them in this paper. The first negative system of the ${N}_{2}^{+}$ spectrum (${B}^{2}\,{\Sigma }_{u}^{+}-{X}^{2}\,{\Sigma }_{g}^{+}$) is the main system of ${N}_{2}^{+}$ at wavelength of 391 nm, originated from the transition between the excited states and the ground state of the molecular ${N}_{2\,}^{+}\,\,$ion. Therefore, the radiation intensity of the first negative band head (0, 0) is directly proportional to the excited state population (${N}_{2\,}^{+}\,({B}^{2}\,{\Sigma }_{u}^{+})$).

In nitrogen plasma environment, the population of the electronic excited state (${N}_{2}\,({C}^{3}{\Pi }_{u})$) is mainly a result of direct impact of excited electron, which has higher energy than the threshold excitation, with the ground state (${N}_{2}\,({X}^{\text{'}}{\Sigma }_{g}^{+})$)^35–39^.

2 $${N}_{2}\,({X}^{\text{'}}{{\rm{\Sigma }}}_{g}^{+})+e\,\to {N}_{2}\,({C}^{3}{\Pi }_{u})+e$$

3 $${N}_{2}\,({C}^{3}{\Pi }_{u})\to {N}_{2\,}^{+}\,({B}^{2}\,{{\rm{\Sigma }}}_{u}^{+})+h\nu $$

Hence, the intensity of emitted photon (356.8) is proportional to the population of ${N}_{2}\,({C}^{3}{\Pi }_{u})$ state^40^.

The transition bands which were observed in the spectra of pure nitrogen and pure acetylene, can also be seen with different intensities in the plasma of the mixture of both gases (Fig. 2c).

The evaluation of the intensity of the emission lines at different applied power yielded an increasing tendency (Fig. 2a). As the emission line intensity is a representative of the species concentration, this result reveals the strong dependence of the species concentration to the plasma power.

The most intense transition lines in the plasma of nitrogen with acetylene are related to ${N}_{2}^{+}$ and ${H}_{\alpha }$, which both transition lines present an increasing trend with increasing power and R. The minimum intensity at the power of 10 W is related to ${H}_{\beta }$, which increases with increasing power.

**Figure 3:**
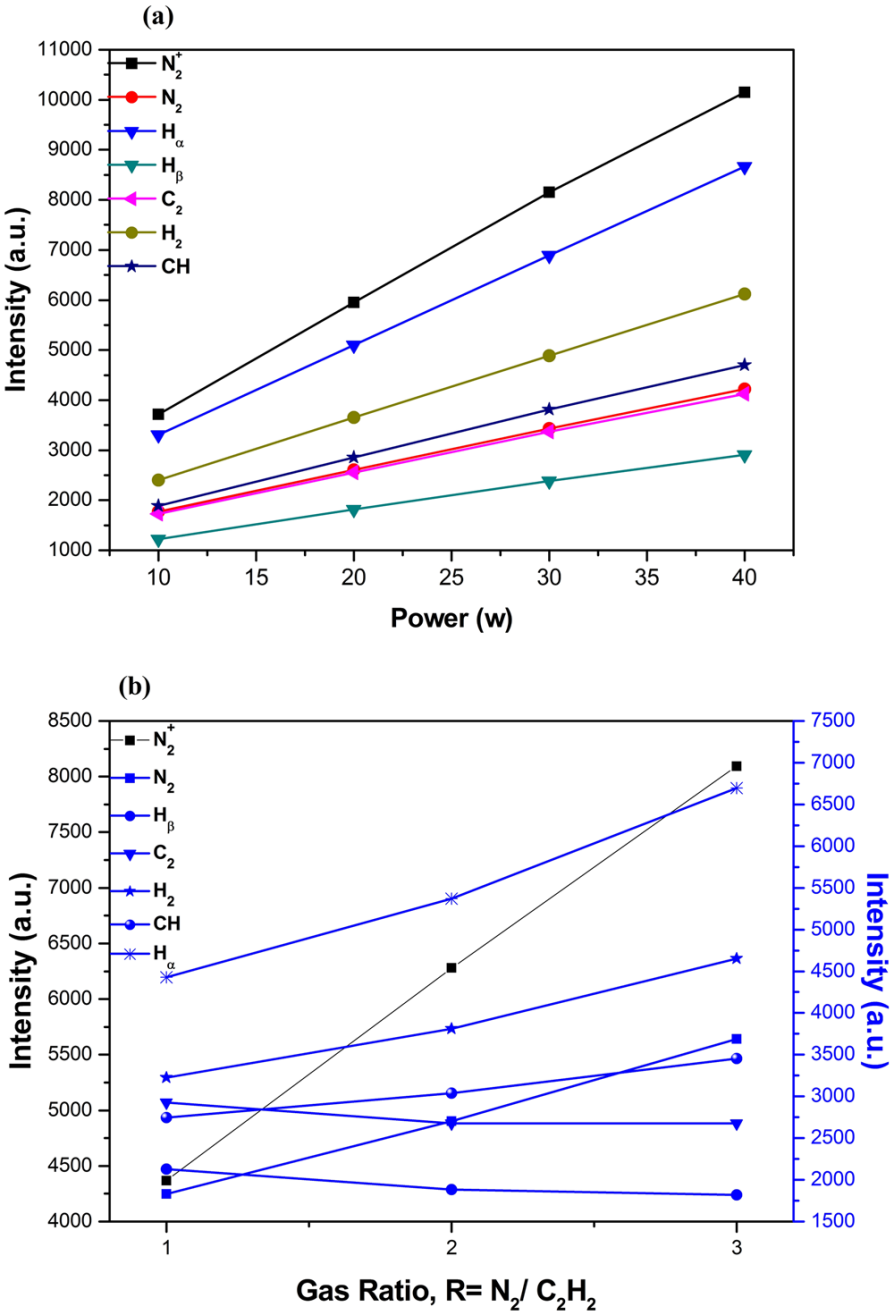
Emission intensities of the main active species as a function of (**a**) applied power in R = 1 and (**b**) gas ratio in P = 20 (w).

Also as is clear in Fig. 3(b), increasing nitrogen flow rate leads to a slight decrease in the intensity of ${{\rm{H}}}_{{\rm{\beta }}}$ and ${{\rm{C}}}_{2}$, while the intensities of other species grow. Nitrogen flow rate increment causes the production of more free radicals in the plasma environment and thereby the probability of combination of those radicals with active carbon-containing groups increases. This leads to a reduction of the intensity of ${{\rm{C}}}_{2}$. The $CN$ band at 388.79 nm was partially overlapped by the (1,1) band of ${N}_{2}$.

### Deposition Rates and Proposed Growth Mechanisms

**Figure 4:**
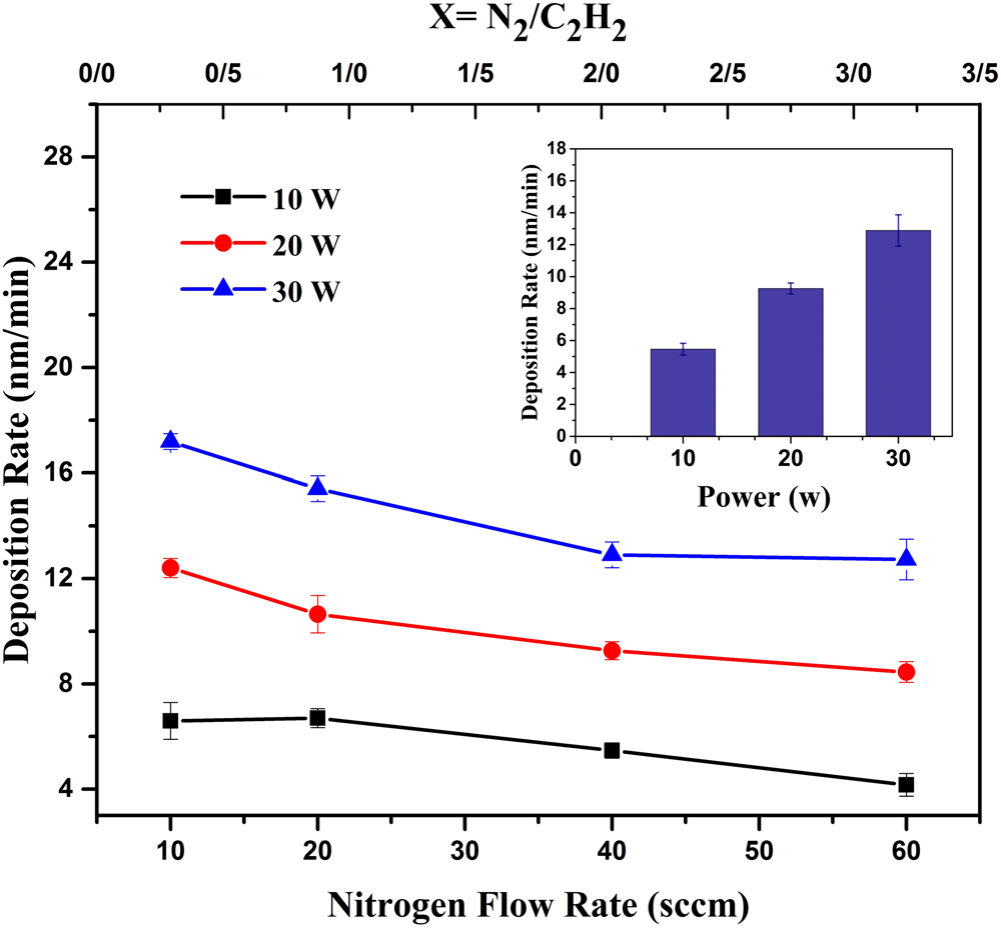
Variation of deposition rate of the L-PPA:N coatings as a function of (**a**) N_2_ gas flow rate in different powers and (**b**) RF power in 40 sccm (C_2_H_2_ flow rate: 20 sccm).

Generally, there are many parameters play important roles in the formation mechanism of acetylene plasma polymerization and nitrogen copolymerization. These parameters are as follows: reactive gases, monomer, pressure, reactor geometry, particles energy, substrate temperature and etc.^41, 42^. The multiplicity of parameters, make the description of polymerization mechanism difficult. However, macroscopically the underlying mechanism consists of passing the gases molecules through the bulk of plasma where the formation of active species and radicals take place. Afterward, the produced monomers and nitrogen containing species recombine and produce stable polymer and copolymer products near the substrate surface in the sheath region. In this process the main reactions consist the interaction between radicals and single reactive site and divalent reactive species^41^. The etching and re-deposition processes on the surface also takes part in plasma copolymerization. The deposition rate (r) is usually determined by the thickness of deposited films (T) as a function of deposition duration time. The deposition rate (r) of the L-PPA:N films as function of N_2_ gas flow rate and applied RF power is shown in Fig. 4.

As is shown in Fig. 4 increasing RF power causes to increase othe thickness of coatings. Power increment leads an increase in decomposition rate of hydrocarbon precursor (acetylene gas) and thereby produces more hydrogen-free radicals, with high cohesion coefficient, on the surface^43^. On the other hand with increasing nitrogen gas flow rate, the thickness of films and also their growth rate decrease. These results can be explained by etching effect of nitrogen atoms^44^.

### Kinetics growth model

As can be seen in Fig. 4 the deposition rate decreases as the nitrogen flow rate increases in the gas mixture. This reduction in deposition rate can be ascribed by the chemical sputtering of the film surface by energetic ${N}_{2}^{+}$ ions bombardment^45^. Indeed, it was found that bombardment of ${N}_{2}^{+}$ ions can sputter carbon atoms with the rate of 0.5 carbon atom per every ${N}_{2}^{+}$ ion^46^. This is also clearly shown in optical emission spectroscopy of the plasma in Fig. 3. In this figure the intensity of ${N}_{2}^{+}$ ion rises more rapidly than other species in the plasma with increasing the nitrogen to acetylene relative flow rate. Increasing the RF power grows the depositor rate of nitrogen copolymerized film. Increasing the plasma power results in the more dissociation of acetylene to carbonaceous species such as C_2_, C-H, C_2_H and … along with the N_2_ molecule to ${N}_{2}^{+}$ ions and etc. The C_2_H radicals, which are the dominant neutral species in acetylene plasma, can easily re-hybridized and produce high density of dangling bonds on the film surface^45^. On the other hand the ${N}_{2}^{+}$ ions chemically sputter the deposited carbon atoms. Therefore, the kinetics growth model of nitrogen copolymerized films can be considered as the competition between deposition and sputtering caused by impinging carbon containing and nitrogen containing species on the film surface. The results show in this competition the deposition process dominates sputtering process with increasing RF power.

### FTIR Spectroscopic Studies

**Figure 5:**
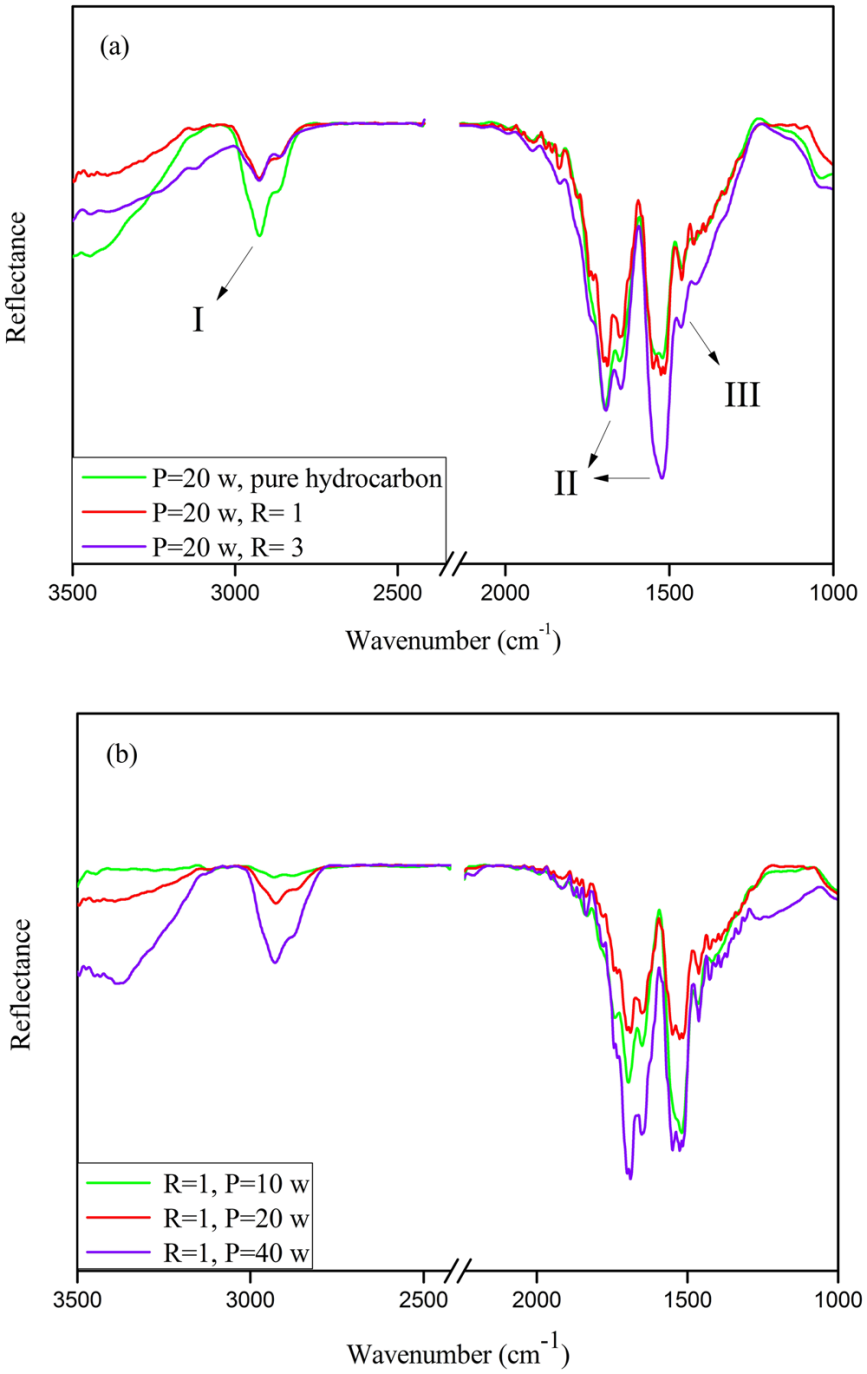
ATR-FTIR spectra of plasma-polymerized deposited in different (**a**) N_2_ flow rates and (**b**) RF power.

Figure 5. illustrates ATR-FTIR spectrum of L-PPA:N surface. According to the spectrum, three bonds were indicated: I. 3000–2800 cm^−1^ C-H stretch; II. 1800–1500 cm^−1^ C=C, C=N stretch; and III. 1500–1300 cm^−1^ CH_2_, CH_3_ bending^47^. Band I comprises a convolution of contributions due to aliphatic CH groups. The assignment of band II is somewhat speculative, the likeliest contributions being C=C or C=N stretching modes^43^. Finally, band III is attributed to disordered highly-ranched aliphatic chains.

As it is observed, band I increases with increasing RF power. However, no regular trend can be detected for II and III, just can be said that in 20 W they have minimum value. Also, increasing nitrogen flow rate leads the increase in II and III, however I shows a negligible change.

### Amine Groups Quantification

Coomassie Brilliant Blue (CBB) Method was used for amine groups’ quantification. CBB measurements as amine groups indexing on the surface of the films were calculated and presented in Table 2. By increasing the nitrogen gas flow rate, the amount of amines increases on the surface of the thin films. Further increasing the gas flow rate leads to a decline in the amount of the surface amines, which can be due to the etching (ablation) effect of nitrogen-containing species. This amount is also decreased by increasing the RF power which is consistent with the results of FTIR. The results show that the low amount of the surface amine groups present in all PPA:N films.

**Table 2:** The amount of CBB absorbed by the L-PPA:N in functions of gas flow rate (R = N_2_/C_2_H_2_) at constant 20 W power and RF power at constant R = 2, respectively.

Gas flow rate (R)	Amount of CBB (ppm)	RF Power (w)	Amount of CBB (ppm)
R = 0.5	$1.33\pm 0.22$	10	$2.21\pm 0.98$
R = 1	$1.67\pm 0.08$	20	$1.93\pm 0.14$
R = 2	$1.93\pm 0.14$	30	$0.84\,\pm 0.31$
R = 3	$1.81\,\pm 082$	—	—

### Surface Wettability and Surface Free Energy

Generally, chemical structure and morphology of the surface control the value of the water contact angle and surface free energy^48^. However, studies reveal that both contact angle and surface free energy are mostly affected by the chemical structure of the surface^49^. Table 3 shows the contact angle values of the films measured about one hour after the deposition as a function of gas flow rate and RF power.

**Table 3:** The contact angle of the untreated and the Plasma - treated BOPP surfaces in functions of gas flow rate (R = N_2_/C_2_H_2_) at constant 20 W power and RF power at constant R = 2, respectively.

Gas flow rate (R)	Contact angle (degree)	Power (w)	Contact angle (degree)
(untreated)	88.27	10	$65.6\pm 0.5$
(pure hydrocarbon)	$75.07\pm 0.3$	20	$64.1\pm 0.4$
R = 1	$67.2\pm 0.16$	30	$63.9\pm 0.6$
R = 2	$64.1\pm 0.4$	40	$62.4\,\pm 0.3$
R = 3	$61.4\,\pm 08$		

**Figure 6:**
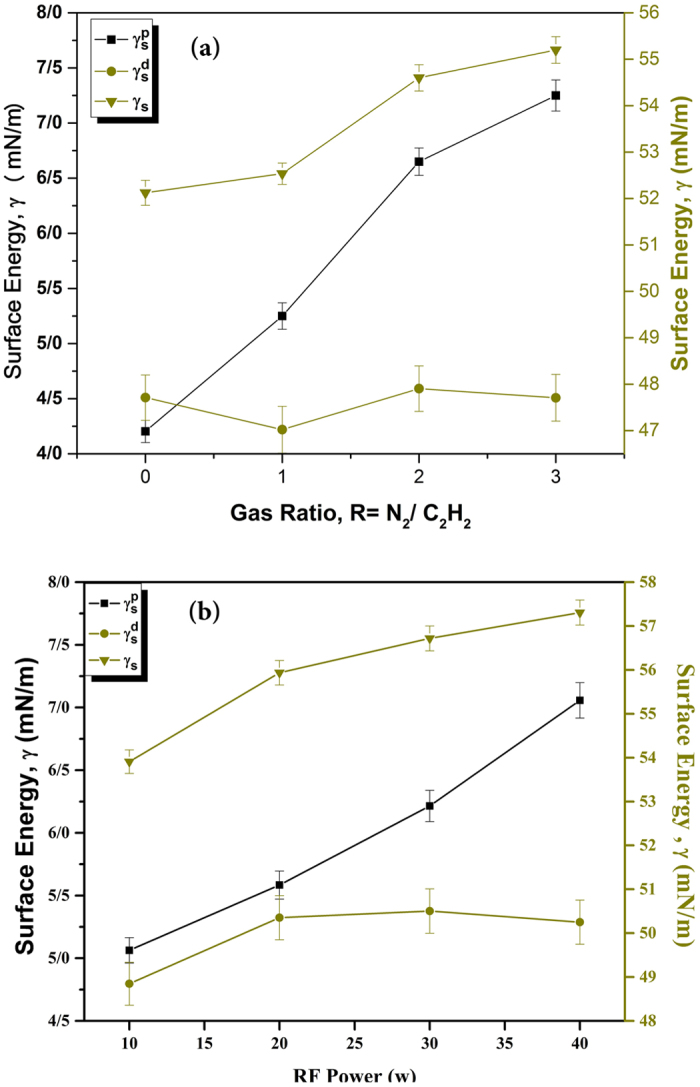
Variation of SFE and its polar and dispersive components in function of (**a**) gas flow rate in R = 1 and (**b**) RF power in 20 (w).

As it can be noticed, wettability of the thin films is increased around 30% after the deposition. Reduction of the contact angle can be explained by the introduction of polar groups onto the surface. This interpretation is corroborated by the surface free energy results, depicted in Fig. 6. This figure shows plots of the total surface energy (${\gamma }_{s}$) determined from Eq. (2), and its polar (${\gamma }_{s}^{p}$) and dispersive (${\gamma }_{s}^{d}$) components as a function of gas flow rate (R) and RF power. The increase in the total surface energy with increasing R and RF power is consistent with the increment of the surface wettability. The ${\gamma }_{s}$ rises monotonically with increasing R, from ∼50 mN.m^−1^ for R = 0 (“pure hydrocarbon”) to ∼55 mN.m^−1^ for R = 3.0. While dispersive component (${\gamma }_{s}^{d}$) decreased slightly, and polar component (${\gamma }_{s}^{p}$) rose considerably on account of the increasing concentration of polar moieties at the films’ surfaces.

According to Fig. 6, increasing power causes the increment of total SFE. Power increment leads to an increase in ion energy that causes the recombination of polymeric chains by ion bombardment in the plasma. This is in good agreement with increasing the reactive species emission lines intensities, which are discussed in OES section. Moreover, increasing ion bombardment energy during the plasma process can slightly raise the temperature of the surface and this can affect the stability of the surface functional groups which are sensitive to temperature. As a result, some of the functional groups (Alkyl, Carboxyl, Amin and …) decay and lower concentration of these groups on the surface causes a decrease of surface hydrophilicity.

### Hydrophobic Recovery of the Surfaces

#### Aging Study in Air

Although after plasma processing the films show an appropriate wettability, the change and mostly decrease in the surface wettability and surface free energy with time is a commonly observed phenomenon. Functional groups at the surface can slowly move and diffuse from the topmost layer to the bulk. This instability of the surface functional groups with time, which is an entropy-driven reorganization of the surface, is known as “hydrophobic recovery”^50^. Hydrophobic recovery leads to the reduction of the surface free energy. Moreover, oxidation of dangling bonds affects the change in surface free energy.

**Figure 7:**
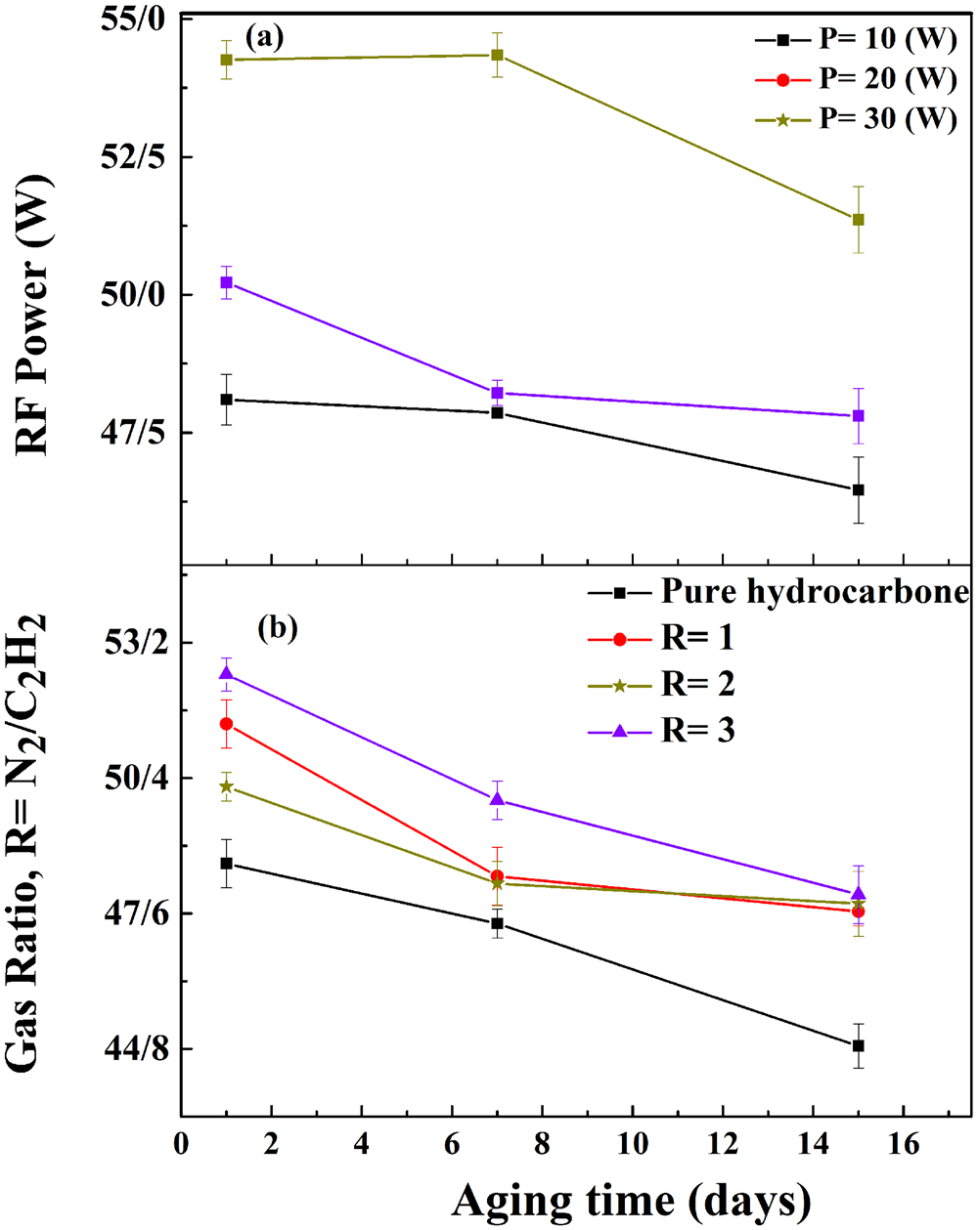
Time evolution of the SFE depending on (**a**) RF power in 20 (w) and (**b**) gas ratio in R = 1.

Plasma-modified polymeric films which were stored in the atmosphere usually indicate a rapid increase in oxygen concentration during the first few days of storage, followed by a slower rise even after months of storage. Some studies also reported a decrease in the ${\gamma }_{s}$ of nitride surface^51^, which accompanied the oxygen uptake. For thin films, aging is more limited 25 due to higher cross-linking and near-uniform composition across the layer, as opposed to plasma modified polymers. However, the same observations such as, oxygen uptake and nitrogen loss, were also reported for N-containing plasma polymers during the first weeks of storage. The physical aging can be easily revealed by repeating contact angle and free energy measurements^52^. To analyze the aging effect, contact angle and surface free energy measurements, using deionized water and diiodomethane liquids, were redid in seventh and fifteenth days after the plasma processing (Fig. 7 (a) and (b)).

#### Aging Study in Liquids

**Figure 8:**
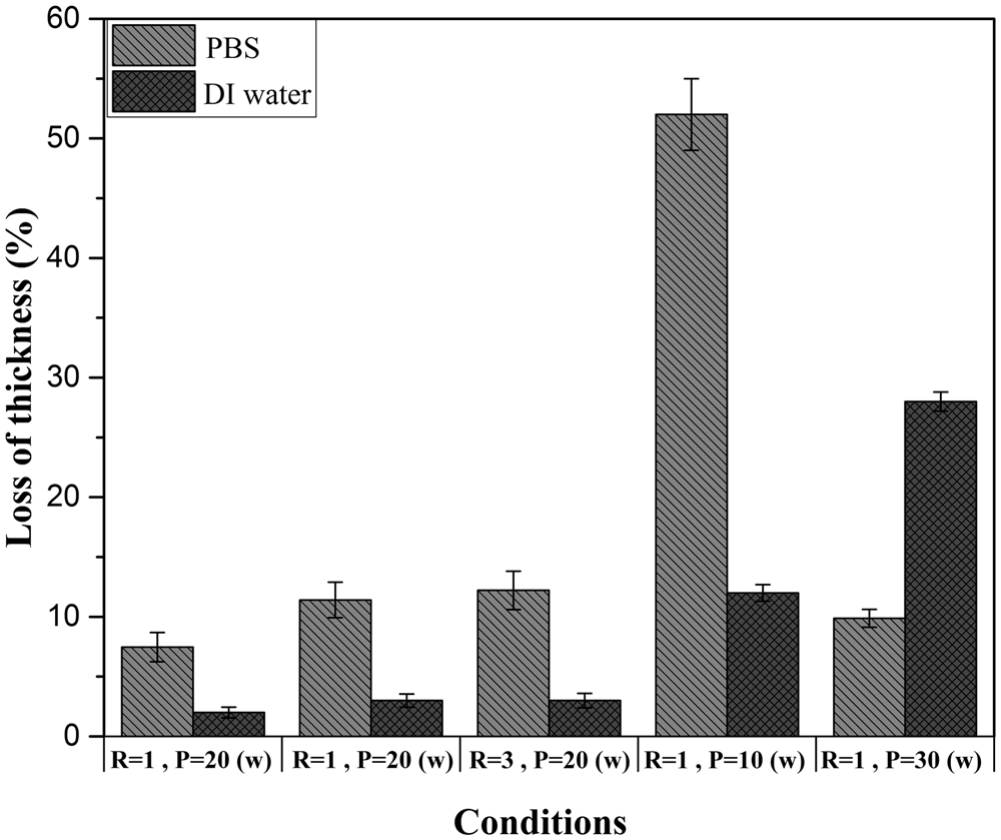
Solubility of deposits, or loss of thickness, after immersion in Milli-Q water and PBS in various condition of plasma deposition.

One of the most essential characteristics of polymeric films for biomedical applications is their insolubility in aqueous environments. Therefore, we assessed the stability of the deposited L-L-PPA:N films on silicon substrates in deionized water (DI) and PBS solution. We measured the thickness of the films and then, put them in the liquid solutions for 24 hours. We checked the possible thickness loss of the films by E.S. measurements (Fig. 8).

By increasing the concentration of nitrogen containing groups on the surface, the solubility increases due to the presence of polar groups and their tendency to polar solvents. On the other hand, the stability is highly affected by cross-linking rate and the presence of oligomer compounds in the surface structure. Figure 8 shows the thickness loss of the samples in different conditions. Increasing R in constant RF power causes a decrease in the stability of L-PPA:N, which is in compliance with Contreras-Garcia *et al*.’s result. Also, as is observed, L-PPA:N has minimum stability in PBS solution in 10 W.

#### Topography of the Surfaces

**Figure 9:**
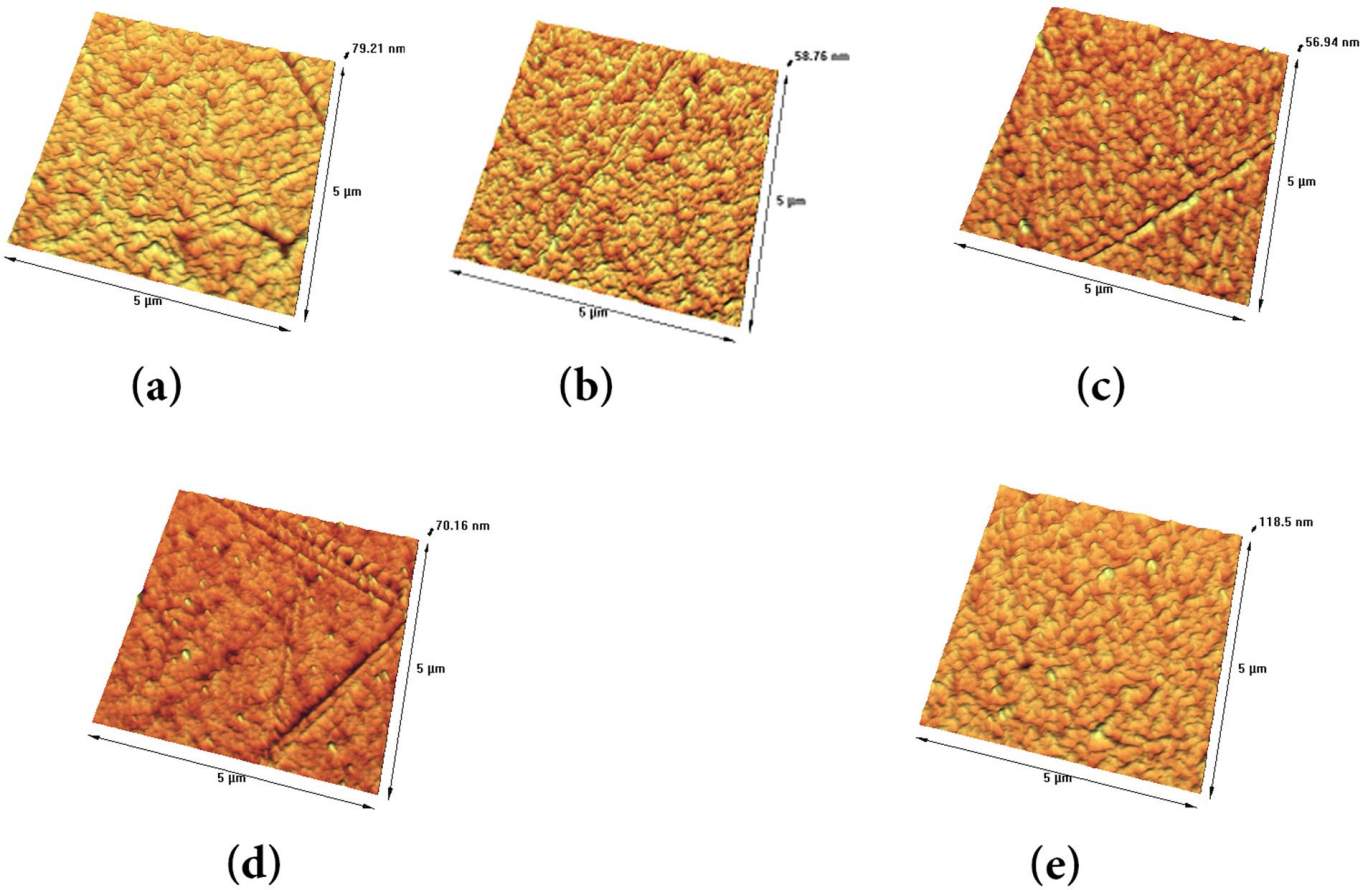
Tree-dimensional AFM images of the plasma-polymers in various (**a**) R = 1, P = 20 (w) (**b**) R = 2, P = 20 (w) (**c**) R = 3, P = 20 (w) (**d**) R = 1, P = 10 (w) (**e**) R = 1, P = 30 (w).

Figure 9 presents the tree-dimensional AFM images of the surface of the thin films under various applied powers and gas mixtures of nitrogen/acetylene. It should be mentioned that in this study acetylene gas flow rate kept constant at 20 sccm in all experiments and the values of root mean square roughness are calculated from 5.00 × 5.00 µm AFM images; the results are presented in Table 4.

**Table 4:** The average roughness of L-PPA:N surfaces in functions of R at constant 20 W power and RF power at constant R = 1.

Gas flow rate (R)	RF Power (watt)	RMS (nm)	R_a_ (pm)
R = 1	20	2.891	467.2
R = 2	20	1.838	254.9
R = 3	20	1.422	197.2
R = 1	10	1.022	240.8
R = 1	30	3.781	524.3

As it can be seen, by increasing nitrogen gas flow rate surface gets smoother, owing to the sputtering effect of the nitrogen ions. The results shows that the roughness values for R = 2 and R = 3 are very close, which can be attributed to the similar intensity of ${H}_{\beta }$ and or ${C}_{2}$ transition lines in the OES spectra of these two cases.

Also, the AFM results illustrate that increasing power roughens the surface. This is in complete compliance with OES results. By increasing power the concentration of reactive species responsible for the etching increases in the plasma environment. This leads to increment of the roughness value. This may also arise from the deposition of carbon decomposed from acetylene.

**Figure 10:**
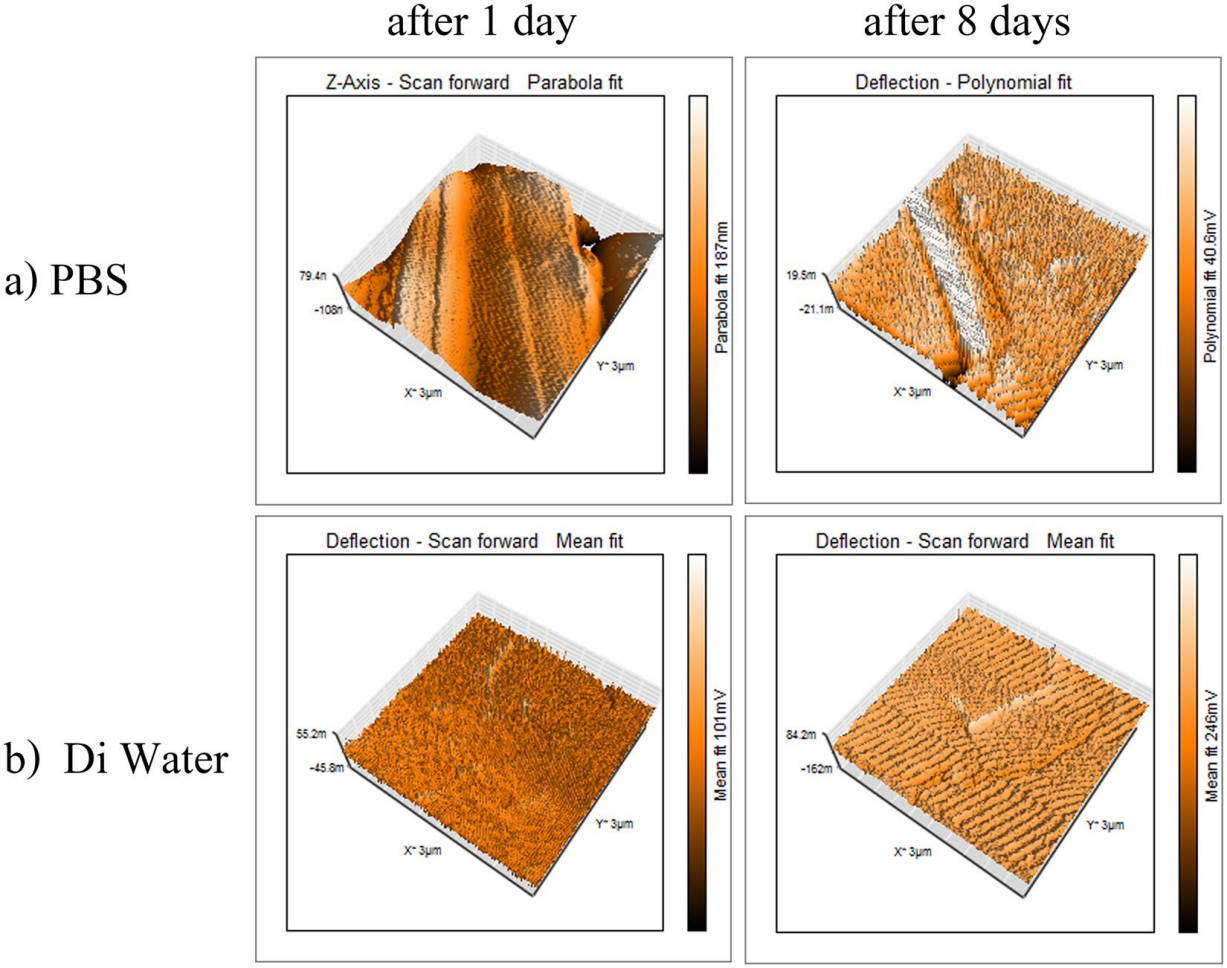
Topology after one and also eight days of immersion into different types of PBS and DI water.

The topology of the specimens are examined after their immersion into PBS and DI water (Fig. 10). It is seen that the topology of the surfaces is completely changed after the immersion.

It can be observed in Fig. 10 that the variations of the topology is more obvious in PBS than that of DI water. In addition, the increment of nitrogen flow increases the instability of the film, which is in agreement with the results of the analysis of the thickness of specimens in liquid medium.

#### Surface potential (zeta potential)

To measure the zeta potential of the surfaces, the apparatus should be calibrated. To do that, PMMA sample was placed into the zeta potential analyzer and after calibration the surface potential of −29.83 mV was obtained. Afterwards, two selected deposited samples (3 hr after deposition) were placed into the analyzer to measure their zeta (surface) potential. The results are summarized in Table 5.

**Table 5:** The zeta (surface) potential for two selected L-PPA:N samples in constant power of 20 W.

	Untreated	R = 1	R = 2
Zeta Potential (mV)	−36.95	−30.13	−28.64

The zeta potential is a description of the electrostatic charge distribution on the surface, hence according to the results it can be said that the samples have negative surface charge (carbon dioxide base films). Regarding the FTIR results this negative surface charge is probably due to the CH bonds and the surface carboxylic groups which present relatively high intensity. It has been confirmed that implant of a controlled negative surface charged (negative zeta potential) materials have promising issues in healing process and tissue regeneration^53^, several studies was also reported that low zeta potential promotes cell attachment, growth and proliferation in comparison to the substrate with no or even positive electric charge^54, 55^. Cells will not interact directly with the surface of substrate or materials in *in-vivo* or *in-vitro* and this attachment is very compliance and dynamic and done in collaboration with many factors^56^.

### Immunofluorescence staining

**Figure 11:**
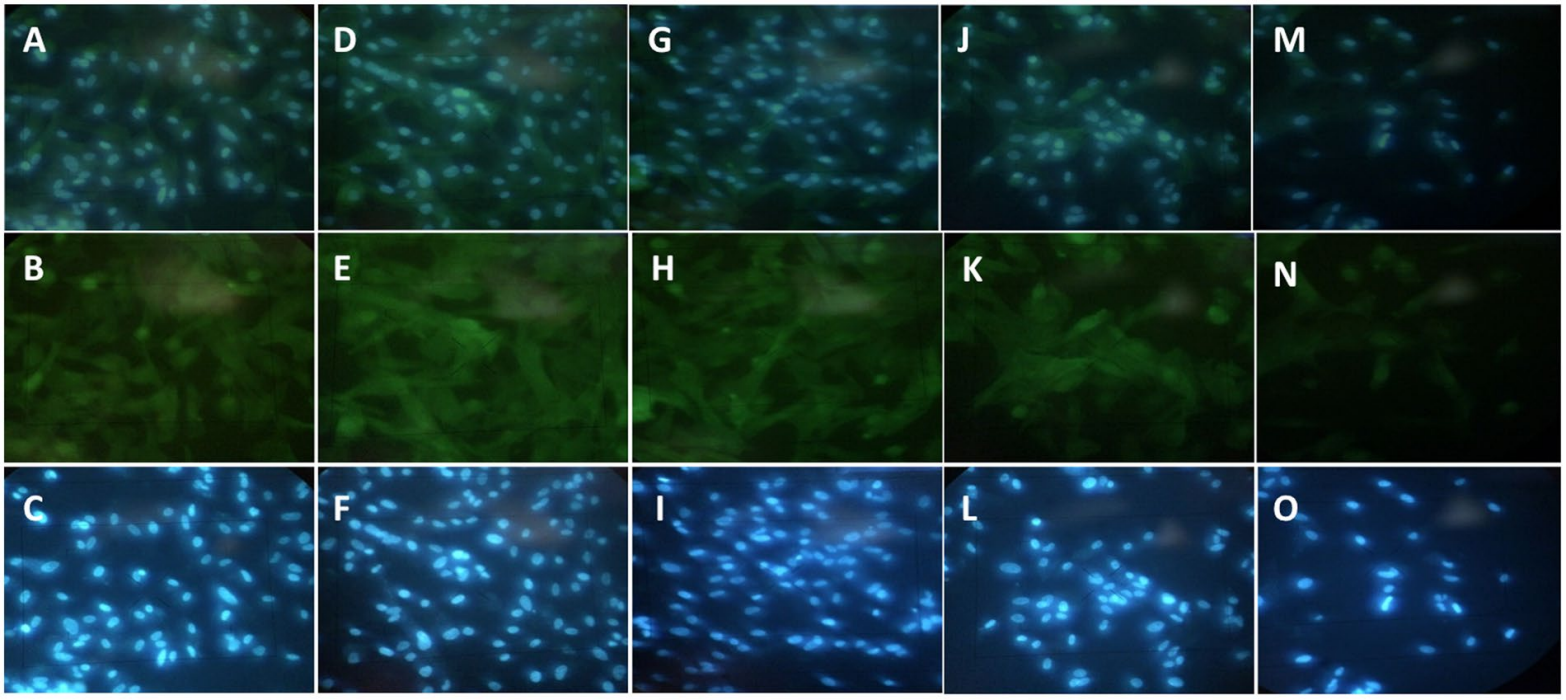
Phalloidin stained stem cells on day 7 after cultured on the surface of plasma treated substrate. (B: P = 10 (W), R = 3; E: P = 20 (W), R = 2; H: 20 (W), R = 1; K: P = 20 (W), R = 2; N: control), stained by DAPI: C: :P = 10 (W), R = 3; F: P = 20 (W), R = 2; I: 20 (W), R = 1; O: control) and Phalloidin and DAPI merged.

Phalloidin staining was used for visualization of the stem cells spindle and fibroblast-like morphology one week after cultured on the surface of different plasma treated substrate, (Fig. 11). Stem cells were absolutely adhered and growth after one week on the surface of plasma treated substrate in comparison to the control plate that very less stem cells attached which demonstrated that biocompatibility of substrate was increased after plasma treatment.

### Real-time PCR

**Figure 12:**
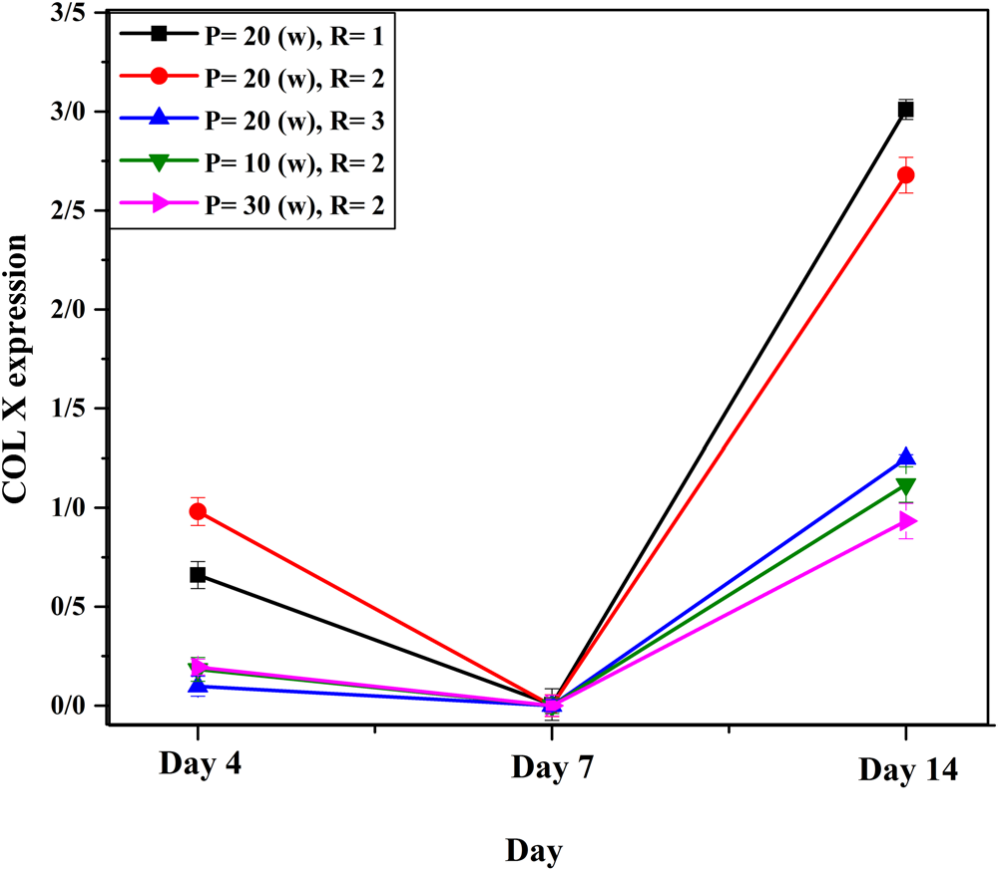
Col X extraction versus day for different L-PPA: N surface.

In our experiments, we analyzed expression of type X collagen, a marker of chondrocyte hypertrophy, in BMSCs incubated in different experimental groups at specific days during 14 days. The Results of real-time PCR for col10 gene have been shown in Fig. 12. The expression of type X collagen mRNA was significantly lower after 4 days of culture, when MSCs were cultured on L-PPA:N, compared to control, suggesting the level of type X collagen mRNA almost returned to control value after 14 days in culture on L-PPA:N surfaces.

As it is shown in Fig. 12, L-PPA:N surfaces caused the col X to be inhibited. The maximum suppression of col X gene expression was monitored in 7th day, whereas in the 14th day L-PPA:N surfaces couldn’t affect col X gene expression any more as it increased again. These results indicated that L-PPA:N coatings may be suitable culture surfaces to inhibit COL10A1 expression in committed MSCs. However, the cellular mechanisms involved in the inhibition of COL10A1 expression by L-PPA:N surfaces are still unknown. Here, we explored the effect of L-PPA:N surfaces on the expression of the type X collagen in MSCs.

In fact, the reduction pattern of type X collagen expression was not observed at day 14 of culture (Fig. 12). Functional groups can be the reason of type X collagen suppression. The results of zeta-potential is in agreement with FTIR and Colorimetric measurements. It seems that the functional groups have a significant role in type X collagen suppression in the early stages of MSCs differentiation; however, the stability of the suppression can be attributed to the amount of the nitrogen functional groups (especially amine groups) as it was reported by Rampersad *et al*.^25^.

## Methods

### Sample Preparation and Deposition of L-PPA:N Coatings

**Figure 13:**
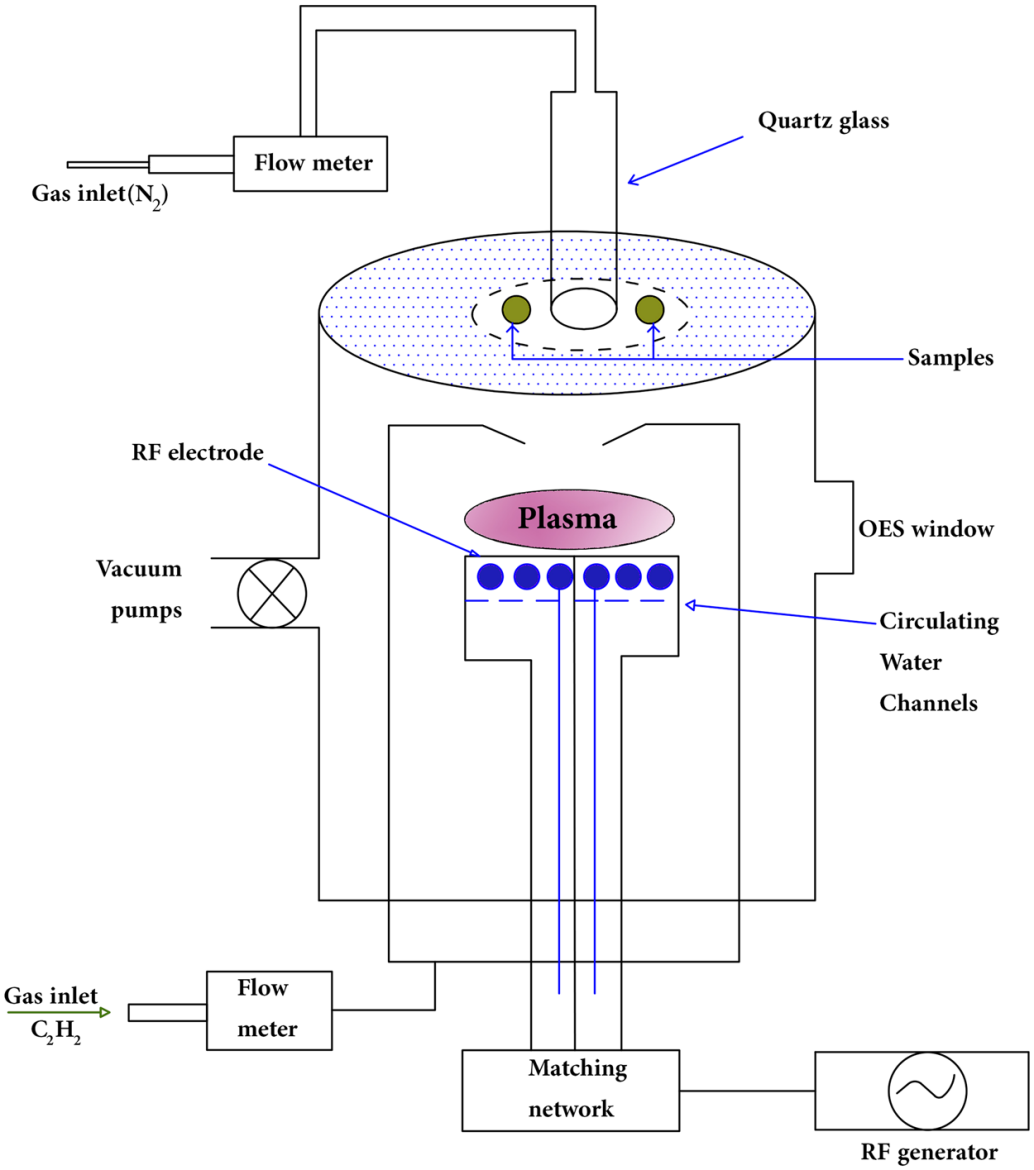
A schematic diagram of plasma polymerization system.

The radio frequency reactor operating at 13.56 MHz in a parallel plate capacitively coupled configuration was used to generate plasma from a mixture of nitrogen (99.9995% purity) with acetylene as monomer (99.6% purity). The plasma reactor is schematically shown in Fig. 13. Biaxially oriented poly (propylene) (BOPP, 50 micron thickness) was the main substrate material for experiments. N-type silicon, PET (100 micron thickness) and steel were also used as substrates in this work.

Samples were washed with soap solution and distilled water, then, sonicated in ethanol bath for 15 min and dried subsequently. The flow rate of acetylene, plasma deposition time and operating pressure were kept constant at 20 sccm, 10 min and 25 mTorr, respectively. In the present work, the effect of nitrogen gas flow rate (0–60 sccm) and applied power (0–30 Watt) on the properties of the coatings were studied.

### Characterization of L-PPA:N Coatings

#### Optical emission spectroscopy

Determining the elemental and molecular composition of plasma environment is one of the methods to estimate the characteristics of the deposited films. One of the most commonly used diagnostic techniques for this purpose is optical emission spectroscopy (OES). In this study, OES was employed for monitoring the plasma and to ensure the plasma reached a steady state after plasma ignition. Characterization of excited species and detection of the optical spectra were performed during the deposition of L-PPA:N films in the wavelength range from 200 to 1100 nm simultaneously by an optical spectrometer (Ocean optics).

#### Deposition Rate

The thickness (T) of coatings was measured by spectroscopic ellipsometry (S.E.) in the ultraviolet–visible (UV–Vis) range using a variable-angle spectroscopic ellipsometer (SE800 DUV, SENTECH Instruments GmbH). Obviously, having knowledge of T permits one to determine deposition rate (r). S.E. measurements were made at the angle of 70 degrees, with 1-nm steps in wavelength, the data being simultaneously fitted using the forouhi bloomer dispersion formula for n. All S.E.-based evaluations were carried out with Spectra Ray software.

#### Infrared Spectroscopy

The surface chemical composition of coatings was determined by attenuated total reflectance furrier-transform infrared (ATR- FTIR) analysis. The ATR-FTIR spectra (ATR- FTIR- Tensor 27, Bruker Co, Germany) were detected in the 3500–1000 cm^−1^ wavenumber, with 100 scans at a resolution of 5 cm^−1^.

### Coomassie Brillant Blue (CBB) colorimetric Method

In this study, quantitative values of amine group density, which influence the MSCs response and collagen-10 suppression^24, 27–29^ were determined via Colorimetric methods. Among commonly used dyes for amine group quantification, Coomassie Brillant Blue (CBB) was selected. The methods reported by Noel *et al*.^57^ were used to quantify the amino group density on LPPA:N films as follow: The thin films were immersed in 1.5 mL of dye solution (0.5 mg/mL) in acidic solution (pH 2.2, 85:10:5 v/v Milli-Q water/methanol/acetic acid) for 5 min at room temperature. The samples were then intensively rinsed with the acidic solution, to remove unbound dye. After drying, the films were immersed in alkaline solution (pH 11.25, 0.125MK2CO3 in 50:50 v/v Milli-Q water/methanol). The pH of the desorbed dye containing solution was adjusted to pH 3 by adding 7.5% v/v of 3 M HCl. The absorbance of the solutions was finally measured at 620 nm.

#### Contact angle analysis and surface free energy

To analyze the hydrophilicity of the L-PPA:N deposited on BOPP surface, water contact angle measurements were carried out. The static water contact angle was measured 3 hours after plasma deposition at room temperature in order to allow all surface relaxation to occur. All measurements were performed using the sessile drop drop technique^58, 59^ with a liquid droplet of a volume of 3 μl. At least three readings were obtained for each sample and the corresponding average values and standard deviations (SDs) were calculated.

The surface free energy (SFE) of a surface is of great importance in different processes. This quantity is determined by the contact angle measurements. In this study, surface energy and its polar (p) and dispersion (d) components were obtained from contact angle data of two liquids, deionized water and diiodomethane (Merck-98%), by using the Owens-Wende method^60, 61^.

4 $${({\gamma }_{s}^{d}{\gamma }_{l}^{d})}^{0.5}+{({\gamma }_{s}^{p}{\gamma }_{l}^{p})}^{0.5}=0.5{\gamma }_{l}(1+\,\cos \,\theta )$$

where, $\theta$, ${\gamma }_{s}^{p}$ and ${\gamma }_{s}^{d}$ are the measured contact angle, polar and dispersion components, respectively. Other parameters are constants which are listed in Table 6 ^61^.

**Table 6:** Test liquids and their surface tension components.

No.	Liquid	Surface tension data (mN/m)		
		${\gamma }_{l}^{d}$	${\gamma }_{l}^{p}$	${\gamma }_{l}$
1	deionized water	21.8	51	72.8
2	Diiodomethane	50.8	0.01	50.81

Finally, the surface free energy was calculated by the following equation:

5 $${\gamma }_{s}=\,(\,{\gamma }_{s}^{d})+\,(\,{\gamma }_{s}^{p})$$

### Ageing effect

To study the stability of the thin films with time, the samples were kept in different environments, air and two different liquids. Deionized water and culture medium (PBS) were selected as two liquid media. For aging analysis in liquid, the thickness of selected samples (coatings on silicon substrate) was first measured. Then, they were placed in the liquid media. After 24 hours the thickness of coatings was measured again using S.E. The L-PPA:N coatings on BOPP were also kept in air for aging analysis. To evaluate the stability of surface properties which were induced by plasma deposition, water contact angle measurements were repeated on the 1st, 7th and 15th days after the deposition.

#### Atomic force microscopy analysis

The surface morphology of the samples was characterized by atomic force microscope made in Iran, modeled ARA/AFM (0103/A) in contact mode which works in ambient conditions. To evaluatethe surface roughness quality, the roughness of samples was derived as a root mean square (RMS) and arithmetic mean (Ra) value from the AFM images with an area of 5.00 × 5.00 µm^2^. Roughness values were determined from three replicates of each sample.

#### Surface potential (zeta)

Electrostatic forces most probably affect the interaction between polymer surface and cell membrane^62^. According to this knowledge, surface potential of deposited plasma-polymers was investigated in this study. The measurement of surface potential of the samples was made 3 hours after plasma deposition using an Electro kinetic Analyzer (EKA Sur PASS–type A, Anton Paar GmbH, Graz, Austria). For this measurement KCl 0.001 M was used as titrant.

### Immunofluorescence staining

On day 7 after cell culture, cells were fixed with 4% paraformaldehyde for 20 minute at 4 °C and then permeabilized by PBS containing either 0.1–0.25% Triton X-100 for 10 minute. Blocking was done by using 3% bovine serum albumin (BSA) for 1 hours at 4 °C. Samples were then incubated with phalloidin-AlexaFluor488 (invitrogen, #A12379) for actin staining at 1:500 for 1 h at 4 °C. After washing with PBS, cells were incubated with DAPI (4′,6-diamidino-2-phenylindole; 1:1000) for nuclear staining.

### Real-time polymerase chain reaction (RT-PCR)

#### Cell culture

Rattus Norvegicus bone marrow Mesenchymal Stem Cell (BM-MSC) line was purchased from the Stem Cell Technology Research Center (Tehran, Iran). For expansion, cells plated into 75 cm^2^ tissue culture flasks, and cultured in Dulbecco’s modified Eagle’s medium (Sigma-Aldrich, USA) supplemented with 10% fetal bovine serum (FBS;, Gibco, UK), with 100 U/ml penicillin, 100 μg/ml streptomycin (Gibco, UK).The cells were in humidified incubator with 5% CO_2_ at 37 °C. Cells were allowed to pass through every 2 to 3 days to maintain a log phase growth. The cell viability was evaluated by trypan blue staining. After the cell cultures reached to an 80% confluence, Cells were dissociated with trypsin (0.25 g/l)/5 mM EDTA (T/E) at 37 °C to obtain single cells, and were plated on the centers of the PPA:N surfaces (covering the entire surface of the regular 24-well culture dishes).The cells were left to adhere to the surfaces for a day. MSCs adhered and grew on the surface.

Media was changed every 2–3 days, up to 14 days in culture. Differentiation toward chondrogenic precursors was evaluated by expression of col 10a1, a marker of chondrocyte hypertrophy, in day 4,7,14 during 2 weeks.

RNA extraction and Real-time polymerase chain reaction (RT-PCR). Total RNA extraction from the bone marrow mesenchymal stem cells (BMSC) was performed using RNXTM-Plus kit (RN7713C; CinnaGen Inc., Tehran, Iran), according to the manufacturer’s instructions. Reverse transcription process was carried out using RevertAid First Strand cDNA Synthesis kit (K1621; Thermo scientific, USA).

The Real Time Quantitative PCR with EvaGreen dye (08-26-00001Solise; Biodyne, Estonia) was

conducted to measure the relative amount of col10a1 gene’s cDNA compare to amount of beta actin’ cDNA as a housekeeping gene in each group. The specific primers which designed utilizing the OLIGO Primer Analysis Software were 5′-AACAGGCAGCAGCACTATG-3′ and 5′-TGAAGCCTGATCCAAGTAGC-3′ for Col10a1 gene while 5′-TATGTTGCCCTAGACTTCG-3′ and 5′-GGTCTTTACGGATGTCAAC-3′ for β-actin gene.

### Statistical analysis

The ANOVA test was performed to test for differences between day and level of COL10A1 expression. Results were considered significant for p < 0.05.

## References

[1] Förch R, Zhang Z, Knoll W (2005). Soft plasma treated surfaces: tailoring of structure and properties for biomaterial applications. Plasma processes and polymers.

[2] Nebe B (2007). Improved initial osteoblast functions on amino-functionalized titanium surfaces. Biomolecular engineering.

[3] Fridman G (2008). Applied plasma medicine. Plasma Processes and Polymers.

[4] Ginsberg GG (2002). The argon plasma coagulatorFebruary 2002. Gastrointestinal endoscopy.

[5] Iseki S (2012). Selective killing of ovarian cancer cells through induction of apoptosis by nonequilibrium atmospheric pressure plasma. Applied Physics Letters.

[6] Fridman, A. *Plasma chemistry*. (Cambridge university press, 2008).

[7] Haack, L., Straccia, A. & Holubka, J. (Google Patents, 2011).

[8] Buckwalter J (2002). Articular cartilage injuries. Clinical orthopaedics and related research.

[9] Martin JA, Buckwalter JA (2002). Aging, articular cartilage chondrocyte senescence and osteoarthritis. Biogerontology.

[10] Buckwalter J, Mankin H (1997). Articular cartilage: degeneration and osteoarthritis, repair, regeneration, and transplantation. Instructional course lectures.

[11] Frenkel SR, Di Cesare PE (2004). Scaffolds for articular cartilage repair. Annals of biomedical engineering.

[12] Burr DB (2004). Anatomy and physiology of the mineralized tissues: role in the pathogenesis of osteoarthrosis. Osteoarthritis and cartilage.

[13] Coutts RD (2001). Matrices for cartilage repair. Clinical orthopaedics and related research.

[14] Sandell LJ, Aigner T (2001). Articular cartilage and changes in arthritis: cell biology of osteoarthritis. Arthritis Research & Therapy.

[15] Cancedda R, Dozin B, Giannoni P, Quarto R (2003). Tissue engineering and cell therapy of cartilage and bone. Matrix Biology.

[16] Bianco P, Robey PG, Simmons PJ (2008). Mesenchymal stem cells: revisiting history, concepts, and assays. Cell stem cell.

[17] Song L, Baksh D, Tuan R (2004). Mesenchymal stem cell-based cartilage tissue engineering: cells, scaffold and biology. Cytotherapy.

[18] He F, Chen X, Pei M (2009). Reconstruction of an *in vitro* tissue-specific microenvironment to rejuvenate synovium-derived stem cells for cartilage tissue engineering. Tissue Engineering Part A.

[19] Poole, A. R., Laverty, S. & Mwale, F. development in the axial and appendicular. *The osteoporosis primer* 3 (2000).

[20] Petit A (2010). Novel insights into the mechanism of decreased expression of type X collagen in human mesenchymal stem cells from patients with osteoarthritis cultured on nitrogen‐rich plasma polymers: Implication of cyclooxygenase‐1. Journal of Biomedical Materials Research Part A.

[21] Brittberg M (1994). Treatment of deep cartilage defects in the knee with autologous chondrocyte transplantation. New england journal of medicine.

[22] Caplan AI (2005). Review: Mesenchymal stem cells: cell–based reconstructive therapy in orthopedics. Tissue engineering.

[23] Mwale F (2006). Suppression of genes related to hypertrophy and osteogenesis in committed human mesenchymal stem cells cultured on novel nitrogen-rich plasma polymer coatings. Tissue Engineering.

[24] Nelea V (2005). Selective inhibition of type X collagen expression in human mesenchymal stem cell differentiation on polymer substrates surface‐modified by glow discharge plasma. Journal of Biomedical Materials Research Part A.

[25] Rampersad S (2011). Stem cells, nitrogen-rich plasma-polymerized culture surfaces, and type X collagen suppression. Tissue Engineering Part A.

[26] Truica-Marasescu F, Girard-Lauriault P-L, Lippitz A, Unger WE, Wertheimer MR (2008). Nitrogen-rich plasma polymers: Comparison of films deposited in atmospheric-and low-pressure plasmas. Thin Solid Films.

[27] Mwale F (2006). The effect of glow discharge plasma surface modification of polymers on the osteogenic differentiation of committed human mesenchymal stem cells. Biomaterials.

[28] Petit A (2011). Effect of nitrogen-rich cell culture surfaces on type X collagen expression by bovine growth plate chondrocytes. Biomedical engineering online.

[29] Girard‐Lauriault PL, Desjardins P, Unger WE, Lippitz A, Wertheimer MR (2008). Chemical Characterisation of Nitrogen‐Rich Plasma‐Polymer Films Deposited in Dielectric Barrier Discharges at Atmospheric Pressure. Plasma Processes and Polymers.

[30] Hosseini S, Sharifian M, Shokri B (2012). Single and dual-mode plasma enhanced chemical vapor deposition of fluorinated diamond-like carbon films. Surface and Coatings Technology.

[31] Rabeau J, John P, Wilson J, Fan Y (2004). The role of C2 in nanocrystalline diamond growth. Journal of Applied Physics.

[32] Akatsuka F, Hirose Y, Komaki K (1988). Rapid growth of diamond films by arc discharge plasma CVD. Japanese journal of applied physics.

[33] Flagan RC, Appleton JP (1972). Excitation Mechanisms of the Nitrogen Frist‐Positive and First‐Negative Radiation at High Temperature. The Journal of Chemical Physics.

[34] Lofthus A, Krupenie PH (1977). The spectrum of molecular nitrogen. Journal of physical and chemical reference Data.

[35] Behringer K, Fantz U (1994). Spectroscopic diagnostics of glow discharge plasmas with non-Maxwellian electron energy distributions. Journal of Physics D: Applied Physics.

[36] Nassar H (2004). N2 + /N2 ratio and temperature measurements based on the first negative N2 + and second positive N2 overlapped molecular emission spectra. Journal of Physics D: Applied Physics.

[37] Petrović ZL, Tochikubo F, Kakuta S, Makabe T (1993). Spatiotemporal Optical Emission Spectroscopy of rf discharges in SF6. Journal of applied physics.

[38] Camacho J, Poyato J, Diaz L, Santos M (2007). Optical emission studies of nitrogen plasma generated by IR CO2 laser pulsesIn Memoriam: Professor Antonio Pardo Martinez. Journal of Physics B: Atomic, Molecular and Optical Physics.

[39] Shaw, M., Ahmad, R., Ikhlaq, U. & Saleem, S. Characterization of Pulsed Dc Nitrogen Plasma Using Optical Emission Spectroscopy and Langmuir Probe.

[40] Qayyum A (2006). Optical emission spectroscopy of the active species in nitrogen plasma. Plasma Devices and Operations.

[41] Hegemann D, Hossain MM, Körner E, Balazs DJ (2007). Macroscopic description of plasma polymerization. Plasma Processes and Polymers.

[42] Yasuda H, Hirotsu T (1977). Some aspects of plasma copolymerization of acetylene with N2 and/or water. Journal of Polymer Science: Polymer Chemistry Edition.

[43] Girard-Lauriault, P.-L. Polymérisation par plasma à pression atmosphérique: caractérisation des dépôts et leurs applications en biotechnologies (2009).

[44] Truica‐Marasescu F, Wertheimer MR (2008). Nitrogen‐Rich Plasma‐Polymer Films for Biomedical Applications. Plasma processes and polymers.

[45] Jacobsohn L, Freire F, Franceschini D, Lacerda M, Mariotto G (1999). Growth kinetics and relationship between structure and mechanical properties of a-C (N): H films deposited in acetylene–nitrogen atmospheres. Journal of Vacuum Science & Technology A: Vacuum, Surfaces, and Films.

[46] Clay K, Speakman S, Amaratunga G, Silva S (1996). Characterization of a‐C: H: N deposition from CH4/N2 rf plasmas using optical emission spectroscopy. Journal of applied physics.

[47] Girard‐Lauriault PL (2005). Atmospheric Pressure Deposition of Micropatterned Nitrogen‐Rich Plasma‐Polymer Films for Tissue Engineering. Plasma Processes and Polymers.

[48] Lawes, G. Scanning electron microscopy and X-ray microanalysis (1987).

[49] Vieu C (2000). Electron beam lithography: resolution limits and applications. Applied Surface Science.

[50] Fritz JL, Owen MJ (1995). Hydrophobic recovery of plasma-treated polydimethylsiloxane. The Journal of Adhesion.

[51] Abbasi-Firouzjah M, Hosseini S-I, Shariat M, Shokri B (2013). The effect of TEOS plasma parameters on the silicon dioxide deposition mechanisms. Journal of Non-Crystalline Solids.

[52] Yun YI (2004). Aging behavior of oxygen plasma-treated polypropylene with different crystallinities. Journal of adhesion science and technology.

[53] Smeets R (2009). A new biphasic osteoinductive calcium composite material with a negative Zeta potential for bone augmentation. Head & face medicine.

[54] Doostmohammadi A (2011). Bioactive glass nanoparticles with negative zeta potential. Ceramics International.

[55] Teng N-C (2001). A new approach to enhancement of bone formation by electrically polarized hydroxyapatite. Journal of dental research.

[56] Scarano A (2006). Maxillary sinus augmentation with different biomaterials: a comparative histologic and histomorphometric study in man. Implant dentistry.

[57] Noel S, Liberelle B, Robitaille L, De Crescenzo G (2011). Quantification of primary amine groups available for subsequent biofunctionalization of polymer surfaces. Bioconjugate chemistry.

[58] Kwok D, Gietzelt T, Grundke K, Jacobasch H-J, Neumann AW (1997). Contact angle measurements and contact angle interpretation. 1. Contact angle measurements by axisymmetric drop shape analysis and a goniometer sessile drop technique. Langmuir.

[59] Rezaei F, Shokri B, Sharifian M (2015). Atmospheric-pressure DBD plasma-assisted surface modification of polymethyl methacrylate: A study on cell growth/proliferation and antibacterial properties. Estuarine, Coastal and Shelf Science.

[60] Żenkiewicz M (2007). Methods for the calculation of surface free energy of solids. Journal of Achievements in Materials and Manufacturing Engineering.

[61] Rezaei F, Abbasi-Firouzjah M, Shokri B (2014). Investigation of antibacterial and wettability behaviours of plasma-modified PMMA films for application in ophthalmology. Journal of Physics D: Applied Physics.

[62] Wertheimer MR (2012). Amine-rich organic thin films for cell culture: possible electrostatic effects in cell–surface interactions. Japanese Journal of Applied Physics.

[63] Jamroz P, Zyrnicki W (2010). Optical emission spectroscopy study for nitrogen–acetylene–argon and nitrogen–acetylene–helium 100 kHz and dc discharges. Vacuum.

[64] Jamroz P, Zyrnicki W (2002). Study of the dc and 100 kHz glow discharges in acetylene-nitrogen mixture by means of optical emission spectroscopy. The European Physical Journal Applied Physics.

[65] Jamroz P, Zyrnicki W (2002). Study of the acetylene–nitrogen, hydrogen–nitrogen and nitrogen with the addition of silicon tetrachloride, 100 kHz low pressure discharge by optical emission spectroscopy. Czechoslovak Journal of Physics.

